# Added Value of Contrast-Enhanced Ultrasound for Cytological Sample Collection of Hepatic and Pulmonary Masses in Dogs

**DOI:** 10.3390/ani16142195

**Published:** 2026-07-15

**Authors:** Manuela Quinci, Nikolina Linta, Pascaline Pey, Marco Baron Toaldo, Andrea Renzi, Francesco Dondi, Alessia Diana

**Affiliations:** 1 Department of Diagnostic Imaging, AniCura Ospedale Veterinario I Portoni Rossi, 40069 Zola Predosa, BO, Italy; quincimanuela@gmail.com; 2 Antech Diagnostics, Mars Petcare Science & Diagnostics, Loveland, CO 80538, USA; 3 Department of Veterinary Clinical Science, University of Bologna, 40064 Ozzano dell’Emilia, BO, Italy; nikolina.linta2@unibo.it (N.L.); f.dondi@unibo.it (F.D.); 4 IMPCOM-MEDICAL S.r.l., 40121 Bologna, BO, Italy; pascaline_pey@hotmail.fr; 5 Division of Cardiology, Clinic for Small Animal Internal Medicine, Vetsuisse Faculty, University of Zürich, CH-8057 Zürich, Switzerland; marco.barontoaldo@uzh.ch; 6 Vedis Laboratory, 4150-367 Porto, Portugal; and.zner@gmail.com

**Keywords:** neoplasia, contrast-enhanced ultrasound, cytological sample collection, veterinary imaging

## Abstract

Ultrasound (US)-guided cytological sample collection (CSC) is widely used for the evaluation of hepatic and pulmonary masses in dogs; however, the presence of intralesional necrosis may reduce diagnostic yield. Contrast-enhanced ultrasonography (CEUS) allows improved assessment of lesion microvascularization and may help identify viable tissue prior to sampling. The aim of this study is to compare the diagnostic yield of CEUS-aided CSC with conventional B-mode US-guided CSC in dogs with hepatic and pulmonary masses. This prospective controlled study with matched controls included 54 client-owned dogs. Twenty-seven dogs underwent CEUS-aided CSC, while 27 matched control dogs underwent conventional B-mode US-guided CSC. Cytological samples were evaluated for diagnostic adequacy, and logistic regression analysis was used to compare groups. No significant difference was found in the probability of obtaining diagnostically adequate samples between techniques. However, CEUS identified non-enhancing areas consistent with presumed necrosis more frequently and with greater extent compared with B-mode US (*p* < 0.05). Although CEUS did not significantly increase overall diagnostic adequacy, it improved identification and delineation of necrotic areas, providing procedurally relevant information for more targeted sampling.

## Simple Summary

Contrast-enhanced ultrasound (CEUS) is an imaging technique that improves visualization of tissue vascularization. In dogs with liver or lung masses, cytological sample collection (CSC) using either fine-needle aspiration or capillary technique is commonly used for diagnosis, but necrotic (non-viable) areas within lesions may reduce sample quality. This study evaluated whether CEUS performed before CSC could improve the identification of viable areas for sampling. Although CEUS did not significantly increase the overall diagnostic yield compared with conventional ultrasound guidance, it enabled better detection and delineation of necrotic regions. This additional information may assist in the selection of optimal sampling sites, particularly in large or heterogeneous lesions. CEUS may therefore represent a useful adjunct tool in the diagnostic evaluation of dogs with hepatic and pulmonary masses.

## 1. Introduction

Ultrasound (US)-guided cytological sample collection (CSC) of abdominal and thoracic organs is a well-established and widely performed diagnostic procedure in small animal practice. The main advantages of conventional B-mode US as a guidance modality include its widespread availability, continuous real-time visualization, and precise needle placement, which together ensure both rapidity and safety of the procedure [1]. Previous studies have reported the diagnostic accuracy of US-guided CSC for abdominal and thoracic lesions in small animals [2,3,4,5]. However, several factors can influence the diagnostic yield of this technique, including the location, size, and histologic nature of the lesion [3,5,6].

Large malignant masses are often associated with extensive areas of necrosis or intralesional hemorrhage [7]. Necrotic regions within a mass are often not reliably identified using conventional US, particularly before liquefaction occurs, potentially resulting in inadequate cytological samples and reduced diagnostic accuracy [7]. Contrast-enhanced ultrasonography (CEUS) using second-generation microbubble contrast agents allows detailed assessment of parenchymal and lesion microvascularization, providing valuable intralesional information to differentiate viable, vascularized tissue from non-viable or avascular areas. On CEUS, non-enhancing regions typically correspond to necrosis or intralesional hemorrhage, whereas hypoenhancing areas may indicate marked fibrosis [8,9,10].

Performing CEUS before US-guided CSC (CEUS-aided CSC) enables identification and avoidance of these non-viable regions during needle placement. In human medicine, CEUS-aided CSC has been shown to improve sampling accuracy, particularly in large neoplastic masses [8,11,12]. Several human studies have demonstrated the superiority of CEUS over conventional US guidance for percutaneous biopsies [8,9].

CEUS may be used either as direct real-time guidance during the procedure or as a preliminary assessment tool to identify the most appropriate target within a lesion [9]. In veterinary medicine, CEUS has been extensively used to evaluate the perfusion of abdominal organs in both healthy and diseased animals. However, data on its role as a guidance tool for cytological sampling or biopsy procedures remain scarce. To date, only a preliminary study has described the feasibility of CEUS-aided biopsy of sentinel lymph nodes in three healthy dogs, demonstrating that CEUS can successfully visualize and guide minimally invasive sampling of lymph nodes draining the mammary glands [13]. Moreover, CEUS performed after renal biopsy has proven useful for detecting post-procedural bleeding and other complications [14].

The aim of the present study is to compare the diagnostic yield of cytological specimens obtained from hepatic and pulmonary masses using CEUS-aided versus conventional B-mode US-guided CSC in dogs. Based on evidence from human medicine, we hypothesized that CEUS performed prior to sampling would improve the diagnostic yield of CSC, particularly in large or heterogeneous masses.

## 2. Materials and Methods

### 2.1. Study Design and Animals

This study was designed as a prospective controlled study with matched controls conducted at the Veterinary Teaching Hospital of the University of Bologna between September 2019 and February 2025. The prospective CEUS-guided arm of the study was approved by the Comitato per il Benessere Animale (COBA), University of Bologna (protocol no. 658), prior to patient enrolment. The study population consisted of client-owned dogs presented for diagnostic evaluation of hepatic or pulmonary masses identified on thoracic or abdominal ultrasonography that were prospectively enrolled in a CEUS-aided CSC group, in which CEUS was performed immediately before CSC to guide needle placement. A matched control group, in which CSC was performed under conventional B-mode ultrasonographic guidance, was retrospectively selected from the hospital archives and included dogs that had undergone conventional B-mode US-guided CSC of hepatic or pulmonary masses between September 2015 and August 2019.

Dogs in the CEUS-aided group were prospectively enrolled if they met the following inclusion criteria: presence of a single hepatic or pulmonary mass confirmed on ultrasonography; performance of CEUS immediately before US-guided CSC to identify the optimal sampling area; availability of cytological evaluation for assessment of diagnostic yield and sample quality. For the purposes of this study, a mass was defined as a lesion with convex margins that distorted the organ contour or internal architecture (e.g., vascular displacement) and measured ≥2 cm in maximal diameter.

For each enrolled dog, the following data were recorded: body weight, body condition score (BCS), body conformation (deep-chested, barrel-chested, or intermediate), breed, anatomic site (liver or lung), and lesion depth within the organ (superficial or deep). Lesion depth was defined as the distance between the cutaneous surface and the target lesion, measured on static ultrasound images using electronic calipers. Masses were classified as superficial when the skin-to-lesion distance was ≤3 cm and as deep when it was >3 cm. Body condition was assessed on physical examination using the 9-point body condition scoring system [15], categorizing dogs as underweight (BCS 1–3), ideal weight (BCS 4–5), or overweight (BCS 6–9). Body conformation (deep-chested, intermediate, or barrel-chested) was determined visually and confirmed according to the dog’s signalment.

Control dogs were cross-matched with those in the CEUS group for body weight, BCS, body conformation, breed, organ involved, and lesion depth. When BCS data were unavailable in the clinical records, they were estimated from thoracic radiographs acquired within one week of the CSC procedure, following the method of described by Linder et al. [16]. The T8 ratio—the ratio between subcutaneous fat thickness and the width of the T8 vertebral body measured on dorsoventral or ventrodorsal projections—was calculated, and BCS was derived using the equation: BCS = 3.056 × log(T8 ratio) + 5.694. Thoracic radiographs were also reviewed to determine body conformation in the control group.

Dogs with deep-seated lesions that could not be reached percutaneously, or with diffuse parenchymal ultrasonographic changes but no discrete mass, were excluded.

### 2.2. Ultrasonographic and CSC Procedures

CEUS was performed in all dogs of the CEUS-aided group immediately before CSC to evaluate lesion perfusion, identify the most viable and suitable area for needle placement, and plan the procedure accordingly. For CEUS and sampling procedures, patients were sedated with an alpha-2 adrenergic agonist (medetomidine or dexmedetomidine) in combination with an opioid (butorphanol, fentanyl, or methadone), according to the patient’s clinical condition and the anesthetist’s preference. All CEUS examinations were carried out using the second-generation contrast agent SonoVue^®^ (Bracco Imaging S.p.a., Milan, Italy), supplied as a lyophilized powder and reconstituted with 5 mL of saline to form a homogeneous microbubble suspension. The contrast agent was administered intravenously as a 0.05 mL/kg bolus, followed by a 5 mL saline flush. Two injections to allow repeated assessment of lesion perfusion and confirm reproducibility of enhancement patterns were systematically performed in each dog.

Ultrasonographic examinations were performed using two ultrasound systems (EPIQ 5G or iU22; Philips Healthcare, Monza, Italy), equipped with contrast-specific software (Pulse Inversion Harmonic and Power Modulation combined, PMPI) operating at a low mechanical index (0.07). Depending on the lesion depth, a linear probe (L3–12 MHz) or curvilinear probe (C2–9 or C2–5 MHz) was used. All CEUS examinations were performed in split-screen mode, allowing simultaneous visualization of B-mode and contrast-enhanced images. Cine-loops of 120 s were recorded starting from the time of contrast injection and were archived in DICOM format. Lesion perfusion was subjectively assessed in real time, with particular attention to the identification of non-enhancing or poorly enhancing areas, presumed to represent necrosis or hemorrhage. This information was used to select the optimal sampling site, corresponding to the most enhancing and accessible region within the lesion.

All CSCs were performed using standard techniques by two ultrasonographers with more than 15 years of experience in diagnostic imaging (AD, MBT), by a board-certified radiologist (PP), or by a diagnostic imaging resident (MQ) under the direct supervision of one of these experienced clinicians, immediately after completion of CEUS; non-enhancing or poorly enhancing regions were intentionally avoided. The capillary (non-aspiration) technique was preferentially employed in all cases. Aspiration was used only when initial capillary sampling failed to yield adequate material. Fine needles (22–25 G) were utilized, either attached to a syringe or as spinal needles, depending on lesion location (superficial vs. deep-seated). For dogs in the control group, the sampling site was selected according to the B-mode ultrasonographic appearance of the lesion, favoring the shortest and safest pathway while avoiding anechoic regions suggestive of cystic or necrotic areas.

All stored CEUS and B-mode images were subsequently reviewed using ShowCase software (Trillium Technology, Ann Arbor, MI, USA) to describe the ultrasonographic characteristics of hepatic and pulmonary masses. The following B-mode features were evaluated: echotexture, subjectively classified as homogeneous or heterogeneous; echogenicity compared with the surrounding parenchyma; size, measured as maximal diameter using electronic calipers; presence of anechoic or hypoechoic intralesional areas.

For the CEUS studies, the distribution of contrast enhancement was described as homogeneous or heterogeneous during the wash-in, peak, and wash-out phases. The presence of non-enhancing areas (presumed necrosis) was recorded, and, when an anechoic area had already been identified on B-mode imaging, its dimensions were compared between CEUS and B-mode images. Other characteristic findings suggestive of malignant behavior were also documented. Based on veterinary literature, findings suggestive of malignant CEUS behavior for hepatic lesions included the presence of tortuous feeding vessels, rim-like enhancement, and early wash-in and wash-out compared with the surrounding tissue. By contrast, a presumed benign CEUS pattern was characterized by lesions that were isoenhancing relative to the surrounding liver parenchyma at peak enhancement and remained isoenhancing during the wash-out phase [17,18]. For pulmonary masses, these included heterogeneous distribution of contrast medium and disruption of the normal pulmonary vascular anatomy, as opposed to benign pulmonary lesions (e.g., inflammatory lesions), which usually have a homogeneous distribution of contrast with typical pulmonary vessels ramification [19,20].

### 2.3. Cytological Assessment

Immediately after CSC, the collected material obtained by both techniques—CEUS-aided CSC and B-mode US-guided CSC—was placed on glass smears by the radiologists or by the clinician in charge of the case and submitted to the on-site clinical pathology laboratory for processing. Cytological specimens were then reviewed by two investigators (a pathologist and a clinical pathologist), and a consensus cytological interpretation was reached. Both investigators were blinded to the cytological sampling method—CEUS-aided or B-mode US-guided.

Each specimen was classified as either adequate or inadequate for interpretation, based on the following criteria: degree of blood contamination; overall cellularity (nucleated cell count); cell preservation; and presence of necrotic debris. For each parameter, a semiquantitative score was assigned, as summarized in Table 1, following a scoring system adapted from previous literature and the institution’s in-house cytopathological protocol [21]. Whenever possible, all samples deemed adequate for interpretation were used to establish a cytological diagnosis.

### 2.4. Statistical Analysis

Descriptive data were expressed as mean ± standard deviation (SD) for normally distributed continuous variables, median (range) for non-normally distributed continuous variables, and counts or percentages for categorical variables. The probability of obtaining a cytological specimen adequate for diagnostic interpretation using CEUS-aided and conventional B-mode US-guided CSC techniques was evaluated through logistic regression analysis, with calculation of odds ratios (ORs) and 95% confidence intervals (CIs). Statistical significance was set at *p* < 0.05. To compare the size of anechoic areas on B-mode US with non-enhancing areas on CEUS, data distribution was assessed using the Shapiro–Wilk test. As measurements were not normally distributed, they were normalized using a Box–Cox transformation. Data were subsequently reported as medians, and significance was again set at *p* < 0.05. Odds ratios and 95% CIs for diagnostic adequacy were calculated separately for pulmonary and hepatic masses and then for the combined dataset. The proportion of lesions with presumed necrosis was calculated as the percentage of masses exhibiting non-enhancing regions on CEUS relative to the total number of masses evaluated. All statistical analyses were performed using commercially available software (JMP Pro 17, JMP Statistical Discovery LLC, SAS Institute S.r.l., Milan, Italy).

## 3. Results

### 3.1. Study Population

A total of 54 dogs met the inclusion criteria and were enrolled in the study. The CEUS-aided group included 27 dogs (19 with hepatic masses and 8 with pulmonary masses), while the control group consisted of an equal number of dogs (19 with hepatic and 8 with pulmonary masses). The CEUS-aided group comprised 12 mixed-breed dogs. The remaining dogs included two Border Collies, two Argentine Dogos, three Labrador Retrievers, two Pinschers, one Golden Retriever, one Chihuahua, one Bichon Frisé, one Jack Russell Terrier, one Bernese Mountain Dog, and one West Highland White Terrier. Most dogs (25/27) had an intermediate chest conformation, and 2 were classified as deep-chested. The body condition score (BCS) ranged from 5 to 7 among dogs with hepatic masses and from 5 to 6 among dogs with pulmonary masses. Median body weight was 18.6 kg (range: 6.4–38.3 kg) for dogs with hepatic lesions and 21.5 kg (range: 4.7–40.0 kg) for dogs with pulmonary lesions. There were 4 intact females, 11 spayed females, 6 intact males, and 6 neutered males.

The control group consisted of 12 mixed-breed dogs and one each of the following breeds: Jack Russell Terrier, German Shepherd, Doberman Pinscher, Flat-coated Retriever, West Highland White Terrier, American Staffordshire Terrier, Epagneul Breton, Labrador Retriever, Belgian Shepherd, Dachshund, Border Collie, Alaskan Malamute, Pinscher, Maltese, and Cane Corso. This group included 2 intact females, 11 spayed females, 9 intact males, and 5 neutered males. Among these dogs, 25/27 had an intermediate chest conformation, and 2/27 had a deep chest conformation to match the dogs in the CEUS-aided group. Eight dogs were medium-sized, 12 large-sized, and 7 small-sized. The body condition score (BCS) ranged from four to seven among dogs with hepatic masses and from five to six among dogs with pulmonary masses. Median body weight was 24.6 kg (range: 4.4–40 kg) for dogs with hepatic lesions and 27 kg (range: 8–34.0 kg) for dogs with pulmonary lesions.

### 3.2. Ultrasonographic Findings

Most lesions (50/54; 92.6%) were superficially located within the organ (35 hepatic, 15 pulmonary), whereas 4 lesions (3 hepatic, 1 pulmonary) were deep-seated. Median lesion size was 6.0 cm (range: 2.0–16.0 cm) for hepatic masses and 4.85 cm (range: 2.0–20.0 cm) for pulmonary masses. On B-mode ultrasonography, the appearance of masses was variable in both groups. Among hepatic lesions, 24/38 (12 in each group) had a complex, heterogeneous echotexture with mixed echogenicity, while 14/38 (7 in each group) appeared homogeneous. Among pulmonary lesions, 7/16 (4 in the CEUS-aided group and 3 in the control group) were heterogeneous on B-mode ultrasound, while 9/16 (4 CEUS-aided, 5 control) were homogeneous.

**Figure 1:**
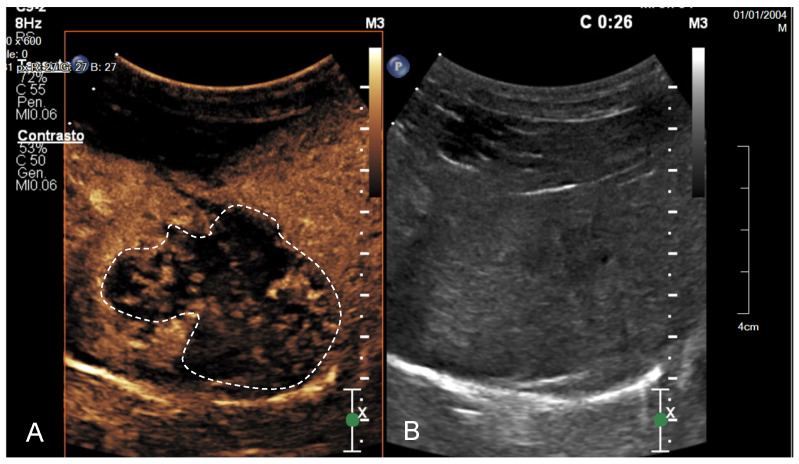
Representative contrast-enhanced ultrasound (CEUS) images of the liver in a dog with a hepatic mass. Images illustrate the contrast-enhanced image on the left (**A**) and the corresponding grayscale image on the right (**B**). During the portal phase, CEUS demonstrates a central hypo/non-enhancing area (white dotted line) suspicious for intralesional necrosis, not appreciable on conventional B-mode ultrasound.

In the CEUS group, 11 hepatic and 6 pulmonary masses exhibited CEUS patterns suggestive of malignant behavior (Figure 1). Pulmonary lesions displayed heterogeneous enhancement with disruption of the normal pulmonary vascular architecture, whereas hepatic masses showed variable arterial enhancement—ranging from hyperenhancement to hypoenhancement—followed by early washout compared with the surrounding parenchyma.

### 3.3. Cytological Analysis

Cytological diagnoses corresponding to hepatic lesions with malignant CEUS patterns included three hepatocellular carcinomas, two well-differentiated hepatic neoplasms (adenoma or carcinoma), one case of nodular hyperplasia or adenoma, and one each of neuroendocrine neoplasia, histiocytic sarcoma, and hemangiosarcoma. In two hepatic cases, cytological diagnosis was not possible due to inadequate samples. Among pulmonary masses showing malignant CEUS behavior, four lesions were cytologically diagnosed as malignant. A complete list of cytological diagnoses, sampling techniques, and corresponding CEUS patterns (benign or malignant) is provided in Table 2 and Table 3.

No significant differences in obtaining adequate cytological samples with CEUS-aided and B-mode US-guided CSC were observed when hepatic and pulmonary lesions were analyzed separately, nor when data from both organs were combined in the univariate logistic regression model. The number of adequate and inadequate samples, together with corresponding *p*-values, is reported in Table 4.

In the CEUS-aided group, 81.5% (22/27) of the samples were adequate and 18.5% (5/27) were inadequate. In the B-mode US-guided group, 85.2% (23/27) of the samples were adequate, and 11.4% (4/27) were inadequate. CEUS identified non-enhancing intralesional areas consistent with presumed necrosis in 62.9% of cases (17/27 masses; 11 hepatic and 6 pulmonary). Among these, 33.3% (9/27 lesions; 7 hepatic and 2 pulmonary) exhibited corresponding anechoic regions on B-mode ultrasonography. The extent of presumed necrotic regions was significantly greater when assessed by CEUS compared with B-mode ultrasonography (*p* < 0.05).

Cases with presumed intralesional necrosis identified on CEUS included the following pulmonary lesions: one adenocarcinoma/carcinoma, one carcinoma, one case of pyogranulomatous and eosinophilic inflammation, one mast cell tumor, and two cases with inadequate samples. Among hepatic lesions with presumed necrotic areas, there were two carcinomas, two lesions classified as adenoma or well-differentiated carcinoma, one hemangiosarcoma, one neuroendocrine neoplasia, one nodular hyperplasia or adenoma, one round cell neoplasia (consistent with possible histiocytic sarcoma), one poorly differentiated neoplasia (suspicious for carcinoma), and two cases with inadequate samples.

Histopathological examination was performed in only two cases. In the CEUS-aided group, one case cytologically interpreted as hepatic degeneration was histologically confirmed as a benign lesion characterized by hepatocellular degeneration and chronic hepatitis. In the control group, one case cytologically diagnosed as hepatic adenoma or well-differentiated carcinoma was histologically confirmed as hepatic adenoma.

## 4. Discussion

The present study evaluates the potential role of contrast-enhanced ultrasonography (CEUS) as an adjunct tool to aid cytological sample collection (CSC) of hepatic and pulmonary masses in dogs. Although no significant difference was found in the probability of obtaining diagnostically adequate cytological samples between CEUS-aided and conventional B-mode US-guided CSC, CEUS provided improved identification and characterization of intralesional non-enhancing areas, consistent with necrosis, which were frequently underestimated or not detected on B-mode ultrasonography. The lack of a statistically significant improvement in diagnostic yield may initially suggest that CEUS-guided CSC does not confer a clear advantage over conventional ultrasonographic guidance. However, this finding should be interpreted in light of several considerations. First, the overall rate of diagnostically adequate cytological samples was relatively high in both groups, potentially limiting the ability to detect incremental improvements. Second, the study population included a heterogeneous spectrum of lesions with respect to ultrasonographic appearance, size, and presumed cytological diagnosis, not all of which were expected to contain extensive necrotic components. Therefore, although the present data do not allow definitive conclusions regarding the lesion types that may benefit most from CEUS guidance, its contribution may be particularly relevant in cases where viable and non-viable tissue coexist, making the identification of appropriate sampling sites more challenging.

Although based on a limited number of cases, most pulmonary and hepatic masses showing presumed necrotic areas on CEUS were cytologically diagnosed as malignant neoplasms. However, the present findings do not allow definitive conclusions regarding the association between CEUS enhancement patterns and lesion malignancy. Importantly, irrespective of lesion type, CEUS enabled improved identification and delineation of presumed non-enhancing areas within lesions compared with conventional B-mode ultrasonography, potentially facilitating the selection of viable tissue for cytological sampling. These findings are partially consistent with previous reports in human medicine showing that CEUS improves visualization of lesion perfusion and intralesional necrosis, thereby facilitating targeting of viable tissue during image-guided tissue sampling [8,9,10,12]. However, direct comparison with the available human literature should be interpreted with caution, as most published studies evaluated CEUS-guided core needle biopsy performed during real-time contrast-enhanced ultrasound examination, whereas in the present study CEUS was used as a pre-procedural planning tool to guide cytological sample collection. This methodological difference, together with the different sampling techniques employed (core needle biopsy versus cytological sampling), may partly explain why the improved delineation of viable tissue observed in the present study did not translate into a statistically significant increase in overall diagnostic yield. The discrepancy observed between CEUS and B-mode ultrasonography in estimating the extent of presumed necrosis likely reflects the limited sensitivity of conventional ultrasonography in detecting early or non-liquefied necrotic changes. From a clinical perspective, the improved characterization of lesion heterogeneity provided by CEUS may still facilitate the selection of appropriate sampling sites, particularly in large or heterogeneous lesions or following previous non-diagnostic sampling attempts. Future studies directly comparing CEUS-assisted cytological sampling with real-time CEUS-guided procedures may further clarify the optimal role of CEUS in interventional veterinary ultrasonography.

The present study has several limitations that should be acknowledged. The sample size was relatively small, particularly when hepatic and pulmonary lesions were analyzed separately, which may have reduced the statistical power to detect differences between groups. Additionally, lesion characterization and selection of sampling sites were based on subjective assessment of CEUS enhancement patterns, which may introduce operator-dependent variability. Although all procedures were performed by experienced operators, the lack of objective perfusion quantification represents a potential source of bias.

The sampling technique was not fully standardized across all cases. Although capillary fine-needle aspiration was preferred, aspiration was used in some cases according to operator preference. Moreover, the number of sampling attempts and prepared smears was not standardized. These factors may have influenced the assessment of cytological sample adequacy and should therefore be considered when interpreting the study findings. In addition, needle gauge was not standardized and was selected according to lesion location, accessibility, and operator preference. Therefore, the possible influence of needle size on cytological sample adequacy could not be assessed and should be considered an additional limitation of the present study.

Interestingly, previous veterinary studies comparing different needle gauges have not demonstrated significant differences in the overall diagnostic quality or diagnostic yield of cytological samples [22,23]. Nevertheless, the potential influence of needle size cannot be excluded in the present study because needle selection was not standardized. Furthermore, this study evaluated the adequacy of cytological samples obtained with CEUS-aided vs. US-guided techniques (i.e., cytological diagnostic yield), whereas diagnostic accuracy of the cytological samples relative to a gold standard diagnosis (e.g., histopathology) was not evaluated. Consequently, samples considered cytologically adequate may not necessarily have been representative of the underlying lesion. For example, aspiration of inflammatory tissue adjacent to necrotic or neoplastic regions could have resulted in adequate cellularity but an inaccurate or misleading cytological diagnosis. Therefore, the results should be interpreted as reflecting sampling adequacy rather than definitive diagnostic accuracy. The study population included a variety of lesion types, and subgroup analysis according to lesion histology or degree of necrosis was not performed. An additional limitation is that cytological sampling was intentionally directed toward presumed viable tissue identified by CEUS, and presumed necrotic areas were not sampled. Consequently, the correspondence between non-enhancing regions on CEUS and cytologically confirmed necrosis could not be verified in the present study. Future prospective studies, including targeted sampling of both viable and presumed necrotic regions, would be valuable to further validate the diagnostic significance of CEUS enhancement patterns.

Despite these limitations, the present findings suggest that CEUS may represent a useful adjunctive imaging technique for identifying presumed non-enhancing areas and guiding CSC in selected hepatic and pulmonary masses in dogs. In particular, CEUS provided additional information regarding intralesional perfusion and presumed necrosis that was not readily obtainable with conventional B-mode ultrasonography. Although no significant improvement in overall cytological diagnostic yield was demonstrated, CEUS improved the delineation of presumed viable tissue and non-enhancing areas, thereby facilitating the selection of appropriate sites for cytological sampling. Although CEUS requires contrast medium administration and increases the cost and duration of the imaging examination, these additional resources may be justified in selected cases in which accurate identification of viable tissue is particularly challenging. Future prospective studies, including procedural time and cost-effectiveness analyses, are warranted to better define the clinical and economic value of CEUS-guided CSC.

## 5. Conclusions

In conclusion, although CEUS-aided CSC did not significantly improve the overall diagnostic yield compared with conventional B-mode US-guided CSC, it improved the detection and delineation of presumed necrotic areas within lesions. These findings suggest that CEUS may assist in identifying appropriate sites for cytological sample collection. Further prospective studies with larger sample sizes and standardized protocols are warranted to better define the role of CEUS in guiding CSC of hepatic and pulmonary masses in dogs.

## Figures and Tables

**Table 1:** Semiquantitative score for cytological analysis. hpf—high power field (40× objective, 10× eyepiece); NA—not applicable.

Parameters	Score			
	0	1	2	3
Blood Contamination	none <2 erythrocytes/hpf	mild >50% erythrocytes in single layer without contact between adjacent cells	moderate >50% erythrocytes in single layer with contact between adjacent cells	severe >50% erythrocytes in multiple layers
Cellularity	scant <10 cells/hpf	low 10–20 cells/hpf	moderate 20–50 cells/hpf	high >50 cells/hpf
Cellular Preservation	scarce <25% preserved cells	moderate 25–50% preserved cells	good >50% preserved cells	NA NA
Necrotic Debris	absent	occasional <25% in 100× field	frequent 25–50% in 100× field	abundant >50% in 100× field
Adequate Sample	no	yes		

**Table 2:** List of hepatic masses with relative cytological sample collection sampling method, adequacy of the obtained smear, cytological diagnosis when applicable. Suspicion of a malignant or benign behavior based on contrast-enhanced ultrasonography (CEUS) pattern is provided for the masses sampled with the CEUS-aided method. NA, not applicable.

Sampling Method	Case Number	Sample Adequacy	Cytological Diagnosis	CEUS Patterns
CEUS-AIDED	1	Adequate	Hepatocellular degeneration	Benign
	2	Adequate	Hepatocellular degeneration	Benign
	3	Adequate	Hepatocellular degeneration	Benign
	4	Inadequate	NA	Benign
	5	Adequate	Neuroendocrine neoplasia	Malignant
	6	Adequate	Carcinoma	Malignant
	7	Adequate	Adenoma or well-differentiated carcinoma	Malignant
	8	Adequate	Hepatocellular degeneration	Benign
	9	Adequate	Hepatocellular degeneration	Benign
	10	Adequate	Hepatocellular degeneration and chronic inflammation	Benign
	11	Adequate	Poorly differentiated malignant neoplasia (suspicion of carcinoma)	Malignant
	12	Inadequate	NA	Malignant
	13	Adequate	Hepatocellular degeneration and possible nodular hyperplasia/adenoma	Equivocal
	14	Adequate	Hepatocellular degeneration and cholestasis	Equivocal
	15	Adequate	Carcinoma	Malignant
	16	Adequate	Nodular hyperplasia or adenoma	Malignant
	17	Adequate	Nodular hyperplasia or well-differentiated neoplasia (adenoma/carcinoma)	Malignant
	18	Adequate	Round cell neoplasm (possible histiocytic sarcoma)	Malignant
	19	Adequate	Hemangiosarcoma	Malignant
US GUIDANCE	20	Adequate	Hepatocellular degeneration	NA
	21	Adequate	Hepatocellular degeneration	NA
	22	Adequate	Adenoma or well-differentiated carcinoma	NA
	23	Inadequate	NA	NA
	24	Adequate	Hepatocellular degeneration and possible fibrosis	NA
	25	Adequate	Carcinoma	NA
	26	Adequate	Adenoma or well-differentiated carcinoma	NA
	27	Adequate	Cholestasis	NA
	28	Inadequate	NA	NA
	29	Adequate	Possible histyocitic sarcoma	NA
	30	Adequate	Adenoma or well-differentiated carcinoma	NA
	31	Adequate	Mesenchymal neoplasm	NA
	32	Adequate	Round cell neoplasia	NA
	33	Adequate	Adenoma	NA
	34	Adequate	Hepatocellular degeneration	NA
	35	Inadequate	NA	NA
	36	Adequate	Neuroendocrine neoplasia	NA
	37	Adequate	Carcinoma	NA
	38	Adequate	Carcinoma	NA

**Table 3:** List of pulmonary masses with relative cytological sample collection sampling method, adequacy of the obtained smear, cytological diagnosis when applicable. Suspicion of a malignant or benign behavior based on the contrast-enhanced ultrasonography (CEUS) pattern is provided for the masses sampled with the CEUS-aided method. NA, not applicable.

Sampling Method	Case Number	Sample Adequacy	Cytological Diagnosis	CEUS Patterns
CEUS-AIDED	1	Adequate	Carcinoma/adenocarcinoma	Malignant
	2	Adequate	Pyogranulomatous and eosinophilic inflammation	Benign
	3	Inadequate	NA	Malignant
	4	Inadequate	NA	Malignant
	5	Inadequate	NA	Malignant
	6	Adequate	Carcinoma	Malignant
	7	Adequate	Adenocarcinoma	Malignant
	8	Adequate	Mast cell tumor	Malignant
US GUIDANCE	9	Adequate	Granuloma	NA
	10	Adequate	Poorly differentiated neoplasm (sarcoma or carcinoma)	NA
	11	Adequate	Adenocarcinoma	NA
	12	Adequate	Histiocytic sarcoma	NA
	13	Adequate	Adenocarcinoma	NA
	14	Adequate	Adenocarcinoma	NA
	15	Inadequate	NA	NA
	16	Adequate	Adenocarcinoma	NA

**Table 4:** Results of logistic regression analysis, showing the number of adequate and inadequate specimens obtained with the two sampling methods. Significance was set at *p* < 0.05. The two sampling methods did not differ statistically when comparing the number of adequate and inadequate specimens of the hepatic and pulmonary masses independently or combined. CEUS, contrast-enhanced ultrasonography; US, ultrasound.

Sampling Method		Number of Adequate Specimens	Number of Inadequate Specimens	Total Number	*p* Value
CEUS-AIDED	LUNG	5	3	8	0.23
	LIVER	17	2	19	0.65
	Pulmonary and hepatic masses combined	22	5	27	0.71
US GUIDANCE	LUNG	7	1	8	
	LIVER	16	3	19	
	Pulmonary and hepatic masses combined	23	4	27	

## Data Availability

The data presented in this study are available on request from the corresponding author. The data are not publicly available due to privacy restrictions related to client-owned animals.

## Abbreviations

The following abbreviations are used in this manuscript:

CEUS	Contrast-enhanced ultrasonography
CSC	Cytological sample collection
